# Association between the Intrinsically Disordered Protein PEX19 and PEX3

**DOI:** 10.1371/journal.pone.0103101

**Published:** 2014-07-25

**Authors:** Katarina Hattula, Daniel Hirschberg, Nisse Kalkkinen, Sarah J. Butcher, Ari Ora

**Affiliations:** Institute of Biotechnology, University of Helsinki, Helsinki, Finland; University of Copenhagen, Denmark

## Abstract

In peroxisomes, peroxins (PEXs) 3 and 19 are the principal protein components of the machinery required for early peroxisomal biogenesis. For further insight into the interaction of PEX3 and PEX19, we used hydrogen exchange mass spectrometry to monitor conformational changes during complex formation between PEX3 and PEX19 in vitro. Our data showed that PEX19 remained highly flexible during interaction with PEX3. However, we could detect three changes, one each in the N-and C-terminus along with a small stretch in the middle of PEX19 (F64–L74) which became shielded from hydrogen exchange when interacting with PEX3. PEX3 became more protected from hydrogen exchange in the binding groove for PEX19 with only small changes elsewhere. Most likely the N-terminus of PEX19 initiates the binding to PEX3, and then subtle conformational changes in PEX3 affect the surface of the PEX3 molecule. PEX19 in turn, is stabilized by folding of a short helix and its C-terminal folding core permitting PEX19 to bind to PEX3 with higher affinity than just the N-terminal interaction allows. Thus within the cell, PEX3 is stabilized by PEX19 preventing PEX3 aggregation.

## Introduction

Eukaryotic cells have membrane-bound organelles in order to compartmentalize and organize cellular functions. One such organelle is the peroxisome. Its biogenesis is orchestrated by peroxins. In this study, we focus on one particular peroxin complex of PEX3 and PEX19 [1]–[3].

Peroxisomes are single-membrane organelles varying in size, shape, and biochemical content depending on cell type and environmental requirements [4]–[6]. The importance of peroxisomes for normal mammalian development has been shown by their linkage to many inherited diseases. These diseases can be divided in to two groups. The first group is peroxisomal biogenesis disorders (PBD) e.g. Zellweger syndrome and the second group is peroxisomal disorders (PD). PBDs show that peroxisomes have essential functions in lipid metabolism and free radical detoxification. These disorders are linked to mutations in peroxins, a diverse set of conserved proteins essential for peroxisome biogenesis and maintenance. The PDs are caused by single peroxisomal enzyme deficiencies [7]–[15]. Generally, dysfunctional PEXs hamper peroxisomal matrix protein localization without affecting the assembly of the peroxisomal membrane compartment. In humans, a subset of peroxins consisting of PEX3, PEX19 and PEX16 are required for the initial steps of peroxisome biogenesis [16]–[20].

PEX19 is an acidic, extended-shaped, soluble 33-kDa protein with a farnesylated C-terminus. Limited proteolysis and circular dichroism spectra analysis have shown that PEX19 is very flexible, especially at the N-terminus [21]. It is mainly found in the cytosol, but a small fraction resides in peroxisomal membranes [3], [22]–[24]. The localization and cotranslational binding properties of PEX19 suggest that it is a cycling receptor. It is thought to function by first binding peroxisomal membrane proteins (PMPs) in the cytosol to prevent aggregation and then to carry the PMPs to the peroxisomal membrane where the cargo-laden PEX19 interacts with PEX3 resulting in PMP insertion into the membrane [25]–[28]. PEX19 is finally released and recycled back to the cytosol [3], [29], [30]. Interestingly, PEX19 has a large number of interaction partner PMPs, but a C-terminally truncated form of PEX19 that binds PEX3, cannot bind PMP22 and PMP70 [31]. This implies that the binding of PEX3 is unique compared to other PMPs [26]–[28], [32]–[34]. Later findings have shed light on peroxisome biogenesis. *In vitro* experiments have shown that PEX3 and PEX19 are needed for preperoxisomal vesicle budding from the ER [35]. These preperoxisomal vesicles fuse together through the activity of PEX1 and PEX6 in *Saccharomyces cerevisiae*. After fusion, the assembly of the full peroxisomal translocon occurs, and the uptake of peroxisomal enzymes from the cytosol can begin [36], [37].

**Figure 1:**
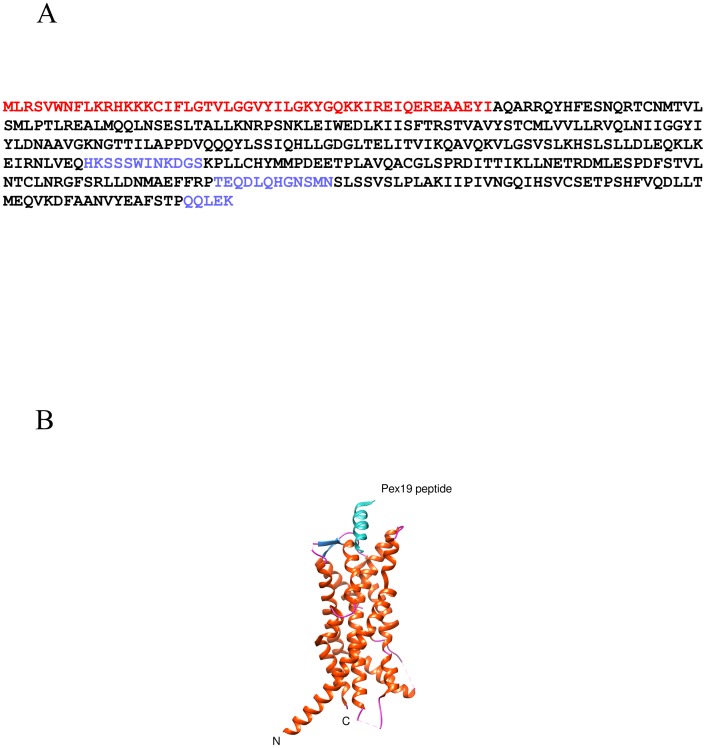
The full length human PEX3 protein sequence. A) The red letters indicate amino acids which are not expressed in the recombinant protein. The black letters indicate amino acids modelled in the X-ray structure (PDB ID: 3AJB) [40]. The blue letters indicate amino acids which are not modelled in the X-ray structure. B) X-ray structure of PEX3 with a PEX19 peptide bound (PDB ID: 3AJB). The secondary structure of PEX3 is colored as a) helix (orange), b) sheet (dark blue) and c) coil (pink). The PEX19 peptide is shown in turquoise.

PEX3 is a 42-kDa, peroxisomal, integral membrane protein. The first 40 N-terminal residues in PEX3 contain both the peroxisomal targeting signal and the single transmembrane domain [18], [38]. The rest of the protein is cytosolic, mediating direct interaction with PEX19. The interaction has been partially characterized through functional domain mapping, pentapeptide insertion screening and by co-crystallization of PEX3 with N-terminal peptides of PEX19 [19], [39]–[41] revealing the atomic detail of this interaction (Figure 1). However, detailed atomic resolution information on the interaction of PEX3 in complex with the full length PEX19 is still missing.

Intrinsically disordered proteins (IDP) are often found to undergo limited folding upon binding and to use flexible regions in partner recognition. Due to their structural flexibility they are often able to carry out several functions [42], [43]. In order to further explore the interaction of just such a protein, PEX19, with one of its interaction partners, PEX3, we utilized a method that would allow us to identify changes in conformation occurring because of the interaction. Hydrogen exchange mass spectrometry (HXMS) reveals the solvent accessibility and stability of local areas in protein samples in solution [44], [45]. In this study we have used HXMS to analyze the interaction between PEX3 and PEX19. Our results establish that PEX19 is extremely dynamic. We found that the N-terminal part of PEX19 binds to PEX3 as previously shown [40],[41]. In addition, we found two protected sites in PEX19 on interaction with PEX3.

## Materials and Methods

### Protein expression and cloning

A cloned human PEX3 in the pET32a vector was a kind gift of Drs. Thilo Stehle and Gabriele Dodt, and has been previously described [41]. The resulting recombinant fusion protein has an N-terminal thioredoxin and His-tag followed by a TEV cleavage site. This soluble PEX3 lacks a functional transmembrane region as the reading frame starts from G25, and has a C235S mutation to improve solubility. For expression of PEX3, *E.coli* Rosetta 2 (DE3) (Novagen) was transformed and grown overnight at 37°C in Luria Bertani (LB) medium supplemented with 100 µg/ml ampicillin (Sigma-Aldrich) and 34 µg/ml chloramphenicol (Sigma-Aldrich). It was then diluted 100-fold in the same medium and incubated under similar conditions until the OD_600_ reached 0.5. The cultures were chilled at 4°C before isopropyl-β-thiogalactopyranoside (IPTG; Fermentas) was added to a final concentration of 0.5 mM. Incubation was continued at 18°C for 16–18 hours before harvesting the cells by low speed centrifugation.

The human PEX19 ORF (Genbank CAG46859.1) was amplified by PCR using an ImageGenes clone as template and the primers: 5′-GAAGAAGAACATATGGCCGCCGCTGAGGAAGG-3′ and 5′-GACGCCCTCGAGTCACATGATCAGACACTGTTC-3′. The PCR product was cloned using Nde I and BamH I sites into the vector pCold I (Takara Bio Inc.) and verified by DNA sequencing. Expression of the full length PEX19 with an N-terminal His-tag was obtained by transforming the plasmid into *E.coli* BL21(DE3) and growing the cells overnight at 37°C in LB medium supplemented with 100 µg/ml ampicillin, diluted 100 fold in the same medium, and incubated under similar conditions until the OD_600_ reached 0.5. The culture was then chilled at 4°C for an hour before IPTG was added to a final concentration of 0.1 mM. Incubation was continued at 15°C for 16–18 h before harvesting the cells by low speed centrifugation.

### Protein purification

PEX3 was purified by resuspending pelleted cells in buffer A (20 mM sodium phosphate, 300 mM NaCl, 5 mM β-mercaptoethanol, 25 mM imidazole, pH 8.0) and lysing with a french press (Thermo Fisher Scientific). After centrifugation at 22 000×g for 30 min at 4°C, the supernatant was collected and incubated with 2 ml Ni-charged beads (IMAC, GE Healthcare) at 4°C, in a head to head rotator for 30 min. The beads were collected by centrifugation and then washed twice with 50 ml buffer A. Tagged protein was eluted with buffer B (20 mM sodium phosphate, 300 mM NaCl, 5 mM β-mercaptoethanol, 500 mM imidazole, pH 8.0) and immediately diluted four-fold with buffer C (20 mM sodium phosphate, 300 mM NaCl, 5 mM β-mercaptoethanol, pH 8.0). The buffer was finally exchanged by ultrafiltration (10K cutoff, Amicon Ultra Millipore) into buffer C. To remove the tag, PEX3 was digested with ProTEV protease (Promega, #V6051). ProTEV buffer (Promega) was added to the slurry of beads in buffer C (1∶1) with 100 units of His-tagged ProTEV protease per ml of Ni-beads. The protein was cleaved at room temperature for 1–2 h, with gentle mixing, and then incubated at 4°C overnight. The supernatant containing PEX3 was retained. A second incubation with Ni-charged beads removed any traces of protease or other contaminants. The buffer was exchanged either by ultrafiltration (10K cutoff, Amicon Ultra Millipore) or by gel filtration on a Superdex 75 10/300 GL column (GE Healthcare) into buffer C. The purified protein was analyzed by SDS-PAGE and mass spectrometry and used within 2–3 days. Protein concentration measurements were done by UV detection at 280 nm, using an extinction coefficient of 25 900 M^−1^ cm^−1^ [46].

PEX19 was purified by resuspending pelleted cells in buffer D (50 mM Tris, 300 mM NaCl, 25 mM imidazole, pH 7.5) to which Pefabloc SC (Sigma-Aldrich) was added to a final concentration of 0.5 mM and lysing the cells by French Press. After centrifugation (22 000×g, 30 min, 4°C), the supernatant was loaded onto a HisTrap column (GE Healthcare). The column was washed with 10 column volumes of the same buffer and the protein eluted with a linear gradient of imidazole (25–500 mM). The peak fractions were pooled and diluted 1∶50 with buffer (50 mM Tris, 1 mM DTT, pH 7.5) for loading onto an anion exchange column (MonoQ FPLC, GE Healthcare). The column was washed with buffer and the protein eluted with a linear NaCl gradient (0 to 1 M). The peak fractions were pooled and the buffer exchanged to 50 mM Tris, 300 mM NaCl, 1 mM DTT, pH 7.5. Finally, the protein was concentrated to 0.4 mM by ultrafiltration (10K cutoff, Amicon Ultra Millipore) and snap frozen in liquid nitrogen and stored at −80°C until required. The protein concentration was measured by UV detection at 280 nm, using an extinction coefficient of 9970 M-1 cm-1 and the purity assessed by SDS PAGE and mass spectrometry.

### Native Mass Spectrometry

PEX3 was incubated with PEX19 in equimolar concentrations (5–10 µM) overnight at 4°C in 20 mM sodium phosphate, 300 mM NaCl, 1 mM DTT, pH 8.0. ESI-MS spectra were recorded on a QToF I (Waters/Micromass). Typically, 10 µl solutions containing the PEX heterodimer were electrosprayed from gold-coated glass capillaries (PicoTip, New Objective). To preserve the non-covalent interactions of the PEX complex, the MS parameters used for the QToF I were: capillary voltage, 1.3–1.8 kV; sample cone, 120 V, analyzer pressure 7–9×10^−5^ mbar; time-of-flight analyzer pressure 7–9×10^−7^ mbar. All spectra were calibrated internally using a solution of cesium iodide (100 mg/mL). Data were processed with MassLynx V4.0 software (Waters/Micromass) with minimal smoothing and without background subtraction [47].

### Amide hydrogen (1H/2H) exchange monitored by mass spectrometry (HXMS)

D_2_O (99.9 atom % D) was obtained from Merck (Darmstadt, Germany). Isotopic exchange was initiated by diluting 5 µl of 200–400 µM of each protein component in 200 µl deuterated PBS buffer (0.14 M NaCl, 2.7 mM KCl, 12.5 mM Na_2_DPO_4_ and 1.7 mM g/l KD_2_PO_4_, pD 7.7 uncorrected value). The exchange was carried out on ice for 0 min, 3 min, 7 min, 40 min and 60 min. The exchange was quenched by adding 8 µl 2.5% TFA. As the PEX complexes precipitated upon freeze/thawing, the samples were analyzed immediately rather than snap-freezing. Water was used for the non-deuterated sample. To determine the degree of back-exchange in the system, fully deuterated protein was prepared by incubation of the samples in 9 M deuterated urea at pH 7.4 overnight at 25°C. Samples were prepared in duplicate.

The liquid chromatography (LC) setup was based on a system described previously [48] with the following modifications: Aqueous solution for desalting was delivered by a Pharmacia LKB HPLC pump 2248 while an organic gradient for elution of peptides/proteins was delivered by a Brownlee Labs MicroGradient System (Applied Biosystems). Samples were loaded onto a column packed with immobilized pepsin (Pierce, Rockford, Illinois, USA) and digested for 15 s. The peptic peptides were desalted for 2 min and eluted with a 9 min gradient (for 7 min, from 12% to 40% acetonitrile, 0.05% TFA and an additional 2 min from 40% to 80% acetonitrile, 0.05% TFA). The LC system was coupled to a QToF I (Waters/Micromass) mass spectrometer. Spray voltage was 3.5 kV, cone voltage 40 V, and ion source block temperature 120°C with a desolvation gas flow of 500 l/h at 200°C and nebulizing gas flow of 20 l/h at room temperature. The HXMS data were analyzed by HX-Express [49]. To identify peptic peptides, non-deuterated protein was digested and desalted as described above. The peptic peptides were eluted and collected for Nanoflow-LC-MS/MS analysis, which was carried out with an UltiMate by LC Packings NanoLC coupled to the QToF I. PEX19 and PEX3 peptic peptides were identified on the basis of a comparative database search using the Mascot MS/MS ion search engine [50] (Matrix Science, London, U.K.) on the generated MS/MS spectra. The in-house database consisted of the human wild-type PEX19 protein sequence and the PEX3 protein sequence. The Mascot score was used to select the peptides with the highest signal:noise ratio (the higher the score, the more confident the result). The Mascot identifications of peptides were subjected to an additional manual validation, where the number of assigned fragment ions and their relative intensities and mass accuracies were taken into account. The peptide ion signals were extracted using MassLynx (Waters), smoothed with a Savitsky Golay filter) and centered (four-channel minimum peak width at half-height, 80% centroid top). The average masses were calculated from the peak lists. Both the absolute and relative (to full deuteration control) deuterium contents were calculated for each time point. As duplicate exchange series were acquired for all samples we calculated the mean error of the percentage deuterium incorporation for all the duplicate measurements. This was 1.6%. When peptides overlapped, those with the highest MASCOT score were used. The overlapping peptides also showed that the amount of the relative hydrogen-deuterium exchange was consistent.

## Results

Hydrogen exchange rates detect the internal motions of folded protein, therefore the pattern of deuterium exchange reveals the solvent accessibility and stability of local areas in the protein sample when the protein undergo fluctuations that expose interior sites to water. The experimental setup is to first incubate the protein under native conditions in a deuterated buffer, allowing exchange of amide hydrogens in the polypeptide. The exchange is quenched by lowering the pH, the protein sample is fragmented by pepsin digest and the resulting fragments are separated with HPLC and analyzed by mass spectrometry. Deuterium incorporation into a peptide fragment causes a progressive mass increase, which is followed over time by repeating the experiments, varying the time of incubation in the deuterated buffer. HXMS experiments were carried out in duplicate with high reproducibility, as we had a mean error of only 1.6% with a standard deviation of 4.1% that would incorporate 95% of all of the data. Thus when we report significant changes in the deuterium incorporation due to complex formation, those are cases where the change is greater than 1.6%.

### Determining deuterium uptake profiles for PEX19 and PEX3 monomers

**Figure 2:**
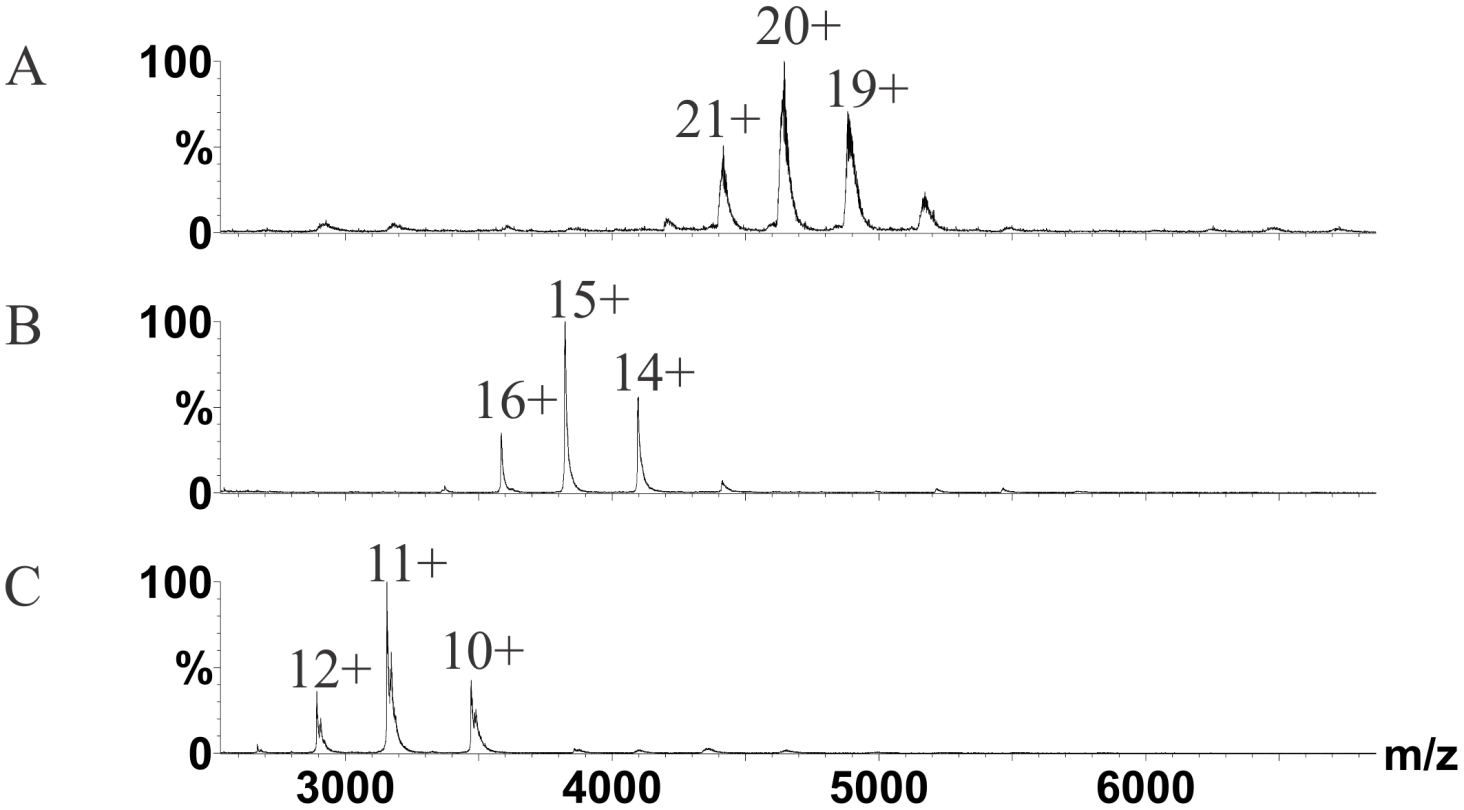
Complex formation of PEX3 with PEX19 monitored by nano-electrospray MS. The spectra were recorded under conditions that preserve non-covalent interactions showing *A*, MS on PEX3T-PEX19 heterodimer, *B*, PEX3 monomer and *C*, PEX19 monomer. Samples were analyzed at 10 µM concentrations for the complex and five-fold higher for the monomers. No homodimers were detected in the spectra. The numbers of the respective peaks represent the charge states. Relative intensities (%) are plotted against mass-over-charge (m/z).

**Figure 3:**
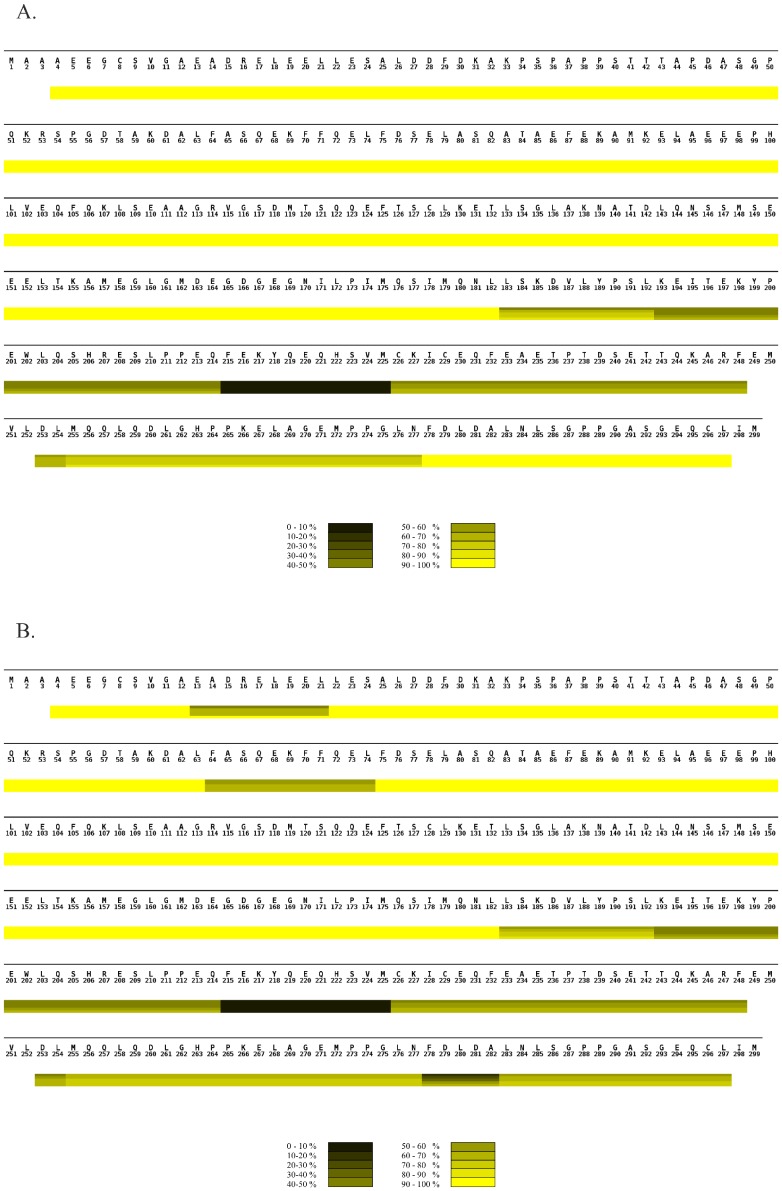
Comparison of hydrogen exchange in PEX19 alone and in complex with PEX3 monitored by MS. (**A**) HXMS heat map of PEX19 monomer summarizing the deuterium uptake over time. (**B**). HXMS heat map of PEX19 in the heterodimer with PEX3 summarizing the deuterium uptake over time. (**A and B**). The sequence of the protein is shown above the heat map. Although the recombinant protein includes an N-terminal tag only the full sequence of human PEX19 is shown with the numbering starting at the initial PEX19 methionine. The heat maps were assembled from individual peptic peptides using MSTools [47]. The extent of the peptide is demarcated by vertical lines in each block, running through all four time point bars, when there is a difference in uptake from the preceding or following peptide. All peptic peptides are shown in **Figure S1**. The scale bar at the bottom of each heat map illustrates the color coding for deuterium uptake as a percentage. The horizontal bars from the top to the bottom in each block of the heat map indicate incubation times of 0, 3, 7, 40 and 60 minutes, respectively. White bars represent the residues for which no data were available.

**Figure 4:**
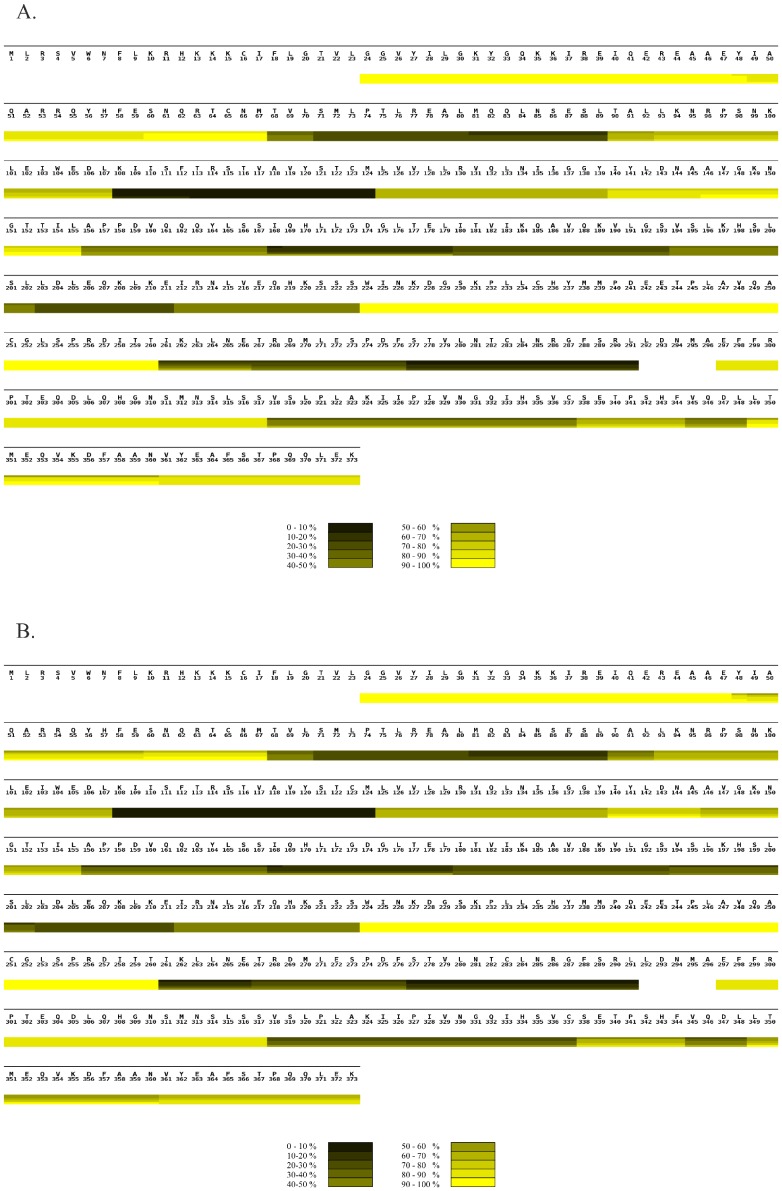
Comparison of hydrogen exchange in PEX3 alone and in complex with PEX19 monitored by MS. A) HXMS heat map of PEX3 monomer summarizing the deuterium uptake over time. B) HXMS heat map of PEX3 in the heterodimer with PEX19 summarizing the deuterium uptake over time. A) and B) The sequence of the PEX3 protein is shown above the heat map. The heat maps were assembled from individual peptic peptides using MSTools [47]. The extent of the peptide is demarcated by vertical lines in each block, running through all four time point bars, when there is a difference in uptake from the preceding or following peptide. All peptic peptides are shown in **Figure S2**. The scale bar at the bottom of each heat map illustrates the color coding for deuterium uptake as a percentage. The horizontal bars from the top to the bottom in each block of the heat map indicate incubation times of 0, 3, 7, 40 and 60 minutes, respectively. White bars represent the residues for which no data were available.

**Figure 5:**
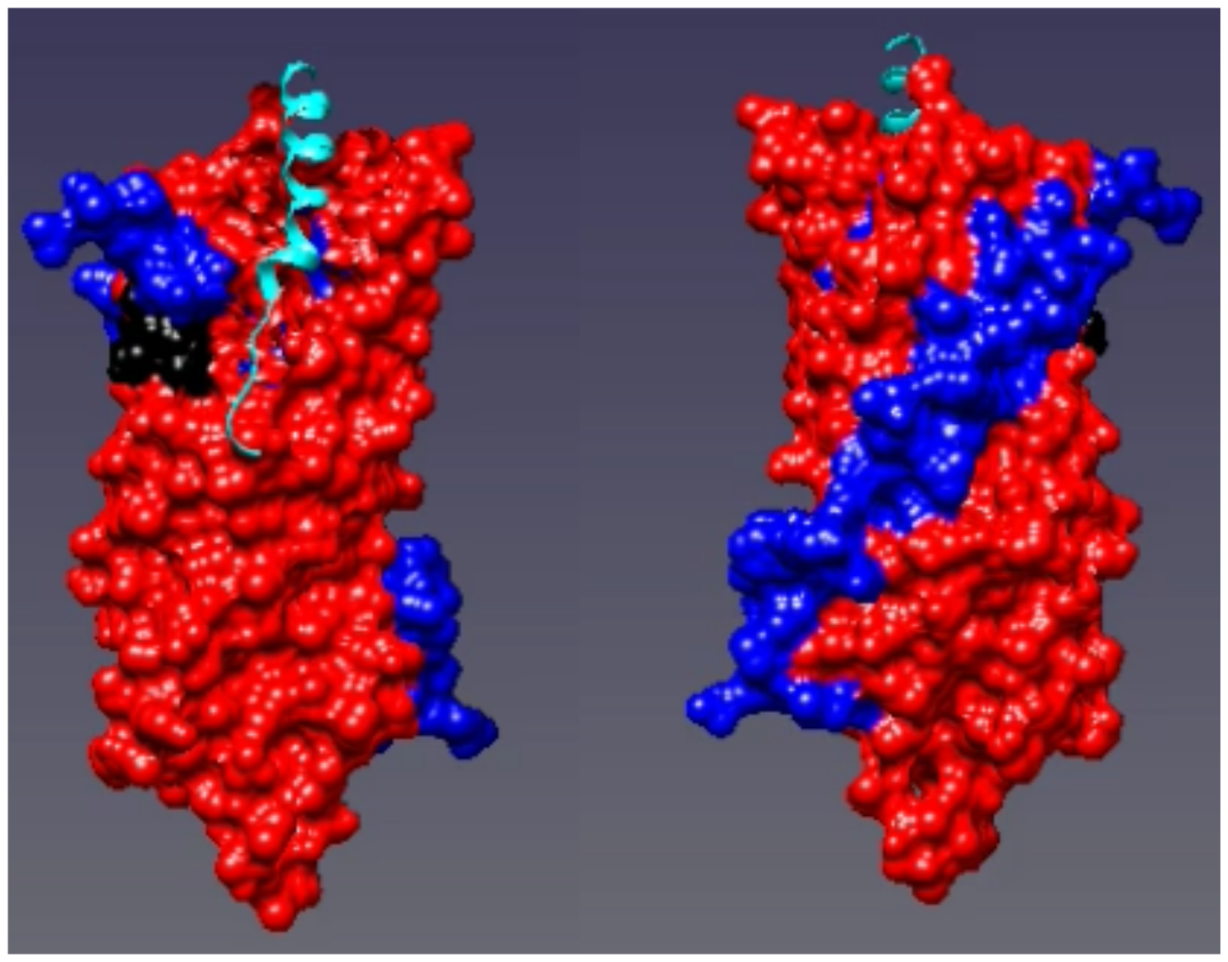
The heat map of PEX3 plotted on the X-ray structure of PEX3 with a PEX19 peptide bound (PDB ID: 3AJB) [40]. Regions where the hydrogen/deuterium exchange decreased in the heterodimer compared to PEX3 alone are shown in red. Regions where the exchange remained the same are shown in blue. The left hand panel is rotated 180° along the Y axis compared to the right hand panel.

We confirmed by native MS that both PEX19 and PEX3 were monomeric under conditions similar to those used for HXMS, but migrated as a 1∶1 complex when mixed (Figure 2). The average masses for PEX19, PEX3 and PEX3:PEX19 complex were 34.8 kDa, 57.4 kDa and 92.2 kDa respectively. These values are a 100 Da bigger than the average calculated mass as expected with native MS as there is a contribution from water and salt adducts. Next, we collected deuterium uptake data from 21 PEX19 and 32 PEX3 peptides, covering 93% and 98% of the protein sequences respectively for the monomers and the complex (Figures 3–5). A heat map of PEX19 (Figure 3A) was rendered from the individual peptic peptide graphs (Figure S1). Where there were several overlapping peptides, exhibiting a similar deuterium uptake profile over time, peptides with the highest signal:noise ratio as defined within the Mascot software [50] were selected to represent the different local regions of PEX3 and PEX19 [47]. The N-terminal part of the recombinant protein, corresponding to the His-tag and the first three amino acids of PEX19, was not detected in the HXMS experiments.

All PEX19 peptides from the N-terminal region A4 - L182, as well as the C-terminal end F278 - L297 were already fully deuterated after 30 seconds incubation in D_2_O (Figure 3A and Figure S1) indicating that these areas, consisting of two thirds of the protein, are highly solvent exposed and structurally disordered [42]. In contrast, the region spanning amino acids L183 - N277 overall showed higher protection against deuterium uptake indicating that it is structurally more ordered, forming a folded core domain overlapping with the region 160–283 which has been shown to crystallize [32].

HXMS on monomeric PEX3 showed a broad range of deuterium uptake rates of the peptic peptides. These rates varied from fully exchanged within 30 s of incubation in deuterated buffer to less than 20% deuterium uptake after 60 min (Figure 4A, Figure S2). Importantly, the local deuterium uptake observed here is in accordance with the recently published structures [40], [41]. For example, the deeply buried α-helix segment spanning K108 - M124 was highly protected while surface exposed and unstructured regions like A250 - T260 had a fast deuterium uptake rate (Figure 4A).

### Monitoring hydrogen exchange of the PEX3/PEX19 heterodimer

We first prepared a PEX3/PEX19 complex by titrating the molar ratio and analyzing the sample by native MS. Uniform complex formation was observed when incubating 5–10 µM PEX3S with 5–10 µM PEX19 at 4°C overnight (Figure 2). This agrees with earlier gel filtration and isothermal calorimetry experiments indicating that the two proteins form a complex [41]. Next we carried out HXMS on the uniform complex as previously described for the monomers. The exchange rates of individual peptides in the monomeric and heterodimeric forms were compared to detect local changes in conformation induced by complex formation.

Three areas with reduced deuterium uptake were found in PEX19 in the complex (Figure 3B). First was the N-terminal spanning region E13 – L21. The N-terminal sequence corresponding to this area has been shown to bind the cytosolic region of PEX3 previously and has been co-crystallized in two previous studies [40], [41]. The other two regions exhibiting reduced deuterium uptake rates are a phenylalanine rich region represented by the F64 - L74 peptide and a stretch of amino acids at the very C-terminus represented by the L183 - L297 peptides (Figure 3B). Notably, peptides in the structured domain of PEX19 (L193 - F248) did not exhibit any significant change in deuterium uptake when PEX19 was bound to PEX3.

We mapped the peptic peptides obtained from the HXMS experiments on the recently solved PEX3 structure [40], [41] and then compared the deuterium uptake profiles of the corresponding peptides from the monomers and the complex. We found several regions on the structure that became more shielded during binding (Figures 4, 5, and Movie S1). One is the apical groove, encompassing peptides L93 - L107, and V318 - C337, that has previously been demonstrated to be a binding site for the N-terminus of PEX19 [40], [41]. Here, we have in addition located a PEX3 area, facing the peroxisomal membrane which is affected by PEX19 binding (Figure 1B). This area contains the peptic peptides A52 - M67, A156 - S167 and I261 - E266 (Figure S2). The results are consistent, shown by the similar behavior of overlapping peptic peptides. However, when we mapped all of the peptides that have any increase in protection on the PEX3 model, virtually the entire surface was slightly protected (Figure 5 and Movie S1).

## Discussion

The focus of this study was to use HXMS to examine interaction sites between PEX3 and PEX19 and to track the structural changes after complex formation. Our data have revealed new insights into likely conformational changes of these proteins *in vitro*. Our results confirmed that the PEX3:PEX19 complex involves the well-established interaction of the PEX19 N-terminus with the apical groove of PEX3.

The N-terminal half (amino acids 1–155) of PEX19 has previously been shown to be highly flexible as it is readily degraded by limited proteolysis [21]. Our HXMS data clearly show a rapid deuterium uptake rate in this area (Figure 3). In addition, our data expanded this area (to 1–182 and 278–297) and demonstrated that as almost two thirds of the protein has such a high uptake rate of deuterium that it can be categorized as an intrinsically disordered protein when alone in solution (reviewed in [51]).

Earlier studies have shown that when the N-terminal region of PEX19 is removed, the protein can still bind PMPs, but no longer localizes to the peroxisomal membranes [19], [25], [26], [34]. Thus the N-terminus of PEX19 is crucial for targeting PEX19 to peroxisomal membranes *in vivo*, presumably via interaction with PEX3. The *in vitro* binding of PEX3 and PEX19 has been studied by surface plasma resonance, microcalorimetry and X-ray crystallography [21], [32], [40], [41], [52]. The affinity of an N-terminal PEX19 peptide was more than 10 fold lower than that for full-length PEX19, implying that there may be more than one binding site between PEX19 and PEX3. The co-crystallisation studies with PEX3 only encompass the N-terminal PEX19 peptide (aa14–33). However, they do indicate that this is the tightest binding site along the PEX19. Here we report lower hydrogen-deuterium exchange in the complex of the two full-length proteins. We compared the exchange in both PEX19 and PEX3 after complex formation. On PEX19, three significant changes occurred. The changes in the N terminus agreed with previous binding affinity assays and crystallography [21], [32], [40]. We saw that the PEX19 region E13-L21 was protected. This corresponds to the PEX19 peptide that has been shown to bind to a hydrophobic groove in PEX3 [40], [41]. A second protected site was seen in a phenylalanine-rich region spanning amino acids F64 - L74. This has the potential to form a helix, as shown by an NMR study of PEX19-PEX14 peptide interactions [53]. Sato *et al.* [40] reported that a Δ45–90 mutant PEX19 can still bind to PEX3 as we would expect for an extended interaction surface. Their pull-down assay did not, however, quantitate the binding affinity. A third region of PEX19 located at the C-terminus (L183–L297), overlapped with the folding core of PEX19 (I171–269) [32].

Addition of PEX19 caused extensive protection of four PEX3 peptides which mapped to the surface of the PEX3 structure at the opposite end of the molecule to the hydrophobic groove. However, mapping of all the increased protection on PEX3 due to PEX19 binding, indicated that there are probably subtle conformational changes affecting most of the PEX3 surface (Figure 5 red surface, and Movie S1).

Based on our MS data we propose the following model for PEX19 binding to PEX3 at the peroxisomal membrane. Most likely the N-terminus of PEX19 initiates the binding to PEX3, and then subtle conformational changes in PEX3 affect the surface of the PEX3 molecule. PEX19 in turn, is stabilized by folding of a short helix, known to be important in PEX14 binding, and its C-terminal folding core permitting PEX19 to bind to PEX3 with higher affinity than just the N-terminal interaction allows. Thus within the cell, PEX3 is stabilized by PEX19 preventing PEX3 aggregation. The binding of PEX19 stabilises the membrane proximal side of PEX3. The folding of the C-terminus of PEX19 on PEX3 binding could affect the observed farnesylation of PEX19 [22].

The intrinsically disordered nature of PEX19, indicated by our HDEX experiments may explain how it can carry out many apparently different roles in the cell [42]. All of the following functions that have been assigned to PEX19 are compatible with it working as an IDP: PEX19 binding to newly translated PMPs in the cytosol, thereby stabilizing them [21], [25]; PEX19 as a membrane insertion factor [33], [54], as a factor for the association and dissociation of protein complexes already in the membrane [34] and as a shuttling receptor [3], [19], [25]. Recent developments in the field of IDP have revealed that although some cooperative folding and binding may be observed in IDP interactions, it can be very limited and does not necessarily involve the whole of the disordered region, instead these flexible linkers offer a platform for multiple other coupling reactions in the cell. IDPs are tightly regulated at all stages, from their synthesis which controls their availability, by post-translational modifications affecting their activity and interactions, to their degradation affecting their overall life time. These measures help to maximize their specificity [55], [56]. We propose that PEX19 regulates many signaling pathways in the cell as an IDP, exemplified by the PEX3:PEX19 interaction.

## Supporting Information

Figure S1 **Relative deuterium uptake of PEX19 peptic peptides plotted over time.** PEX19 monomer is colored in blue (♦), PEX19:PEX3 complex in red (x).
(TIF)

Figure S2 **Relative deuterium uptake of PEX3 peptic peptides plotted over time.** PEX3 monomer is indicated in blue (♦), PEX3 bound to full-length PEX19 in red (▪).
(TIF)

Movie S1 **The heat map of PEX3 plotted on the X-ray structure of PEX3 with a PEX19 peptide bound (PDB ID: 3AJB)** [40] **.** Regions where the hydrogen/deuterium exchange decreased in the heterodimer compared to PEX3 alone are shown in red. Regions where the exchange remained the same are shown in blue.
(MP4)
