# Supplementary figures and images for: Association between the Intrinsically Disordered Protein PEX19 and PEX3
Source: PLoS One. 2014 Jul 25;9(7):e103101. doi: 10.1371/journal.pone.0103101 (PMC4111287; doi:10.1371/journal.pone.0103101)

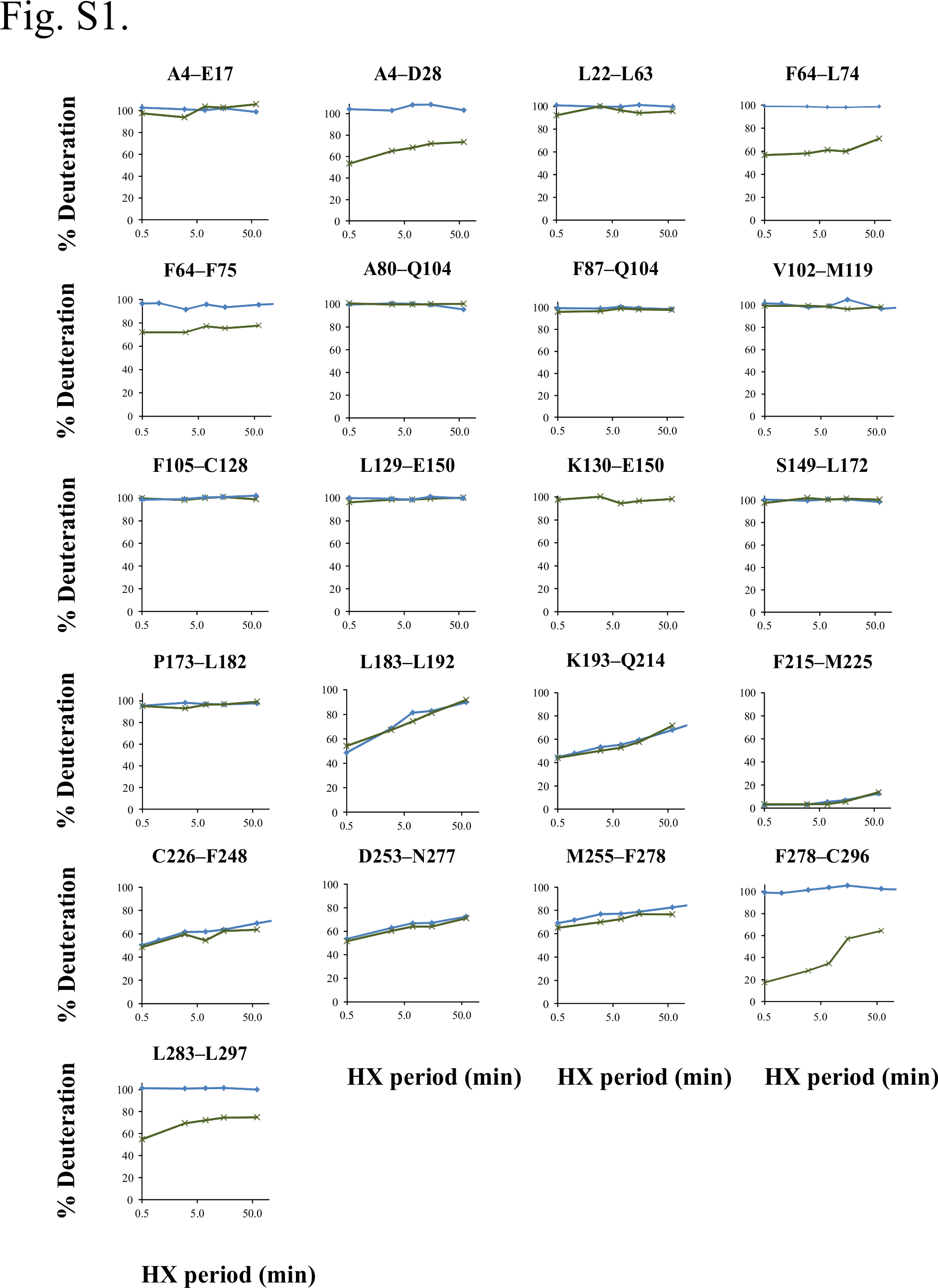

Supplement: Figure S1 — Relative deuterium uptake of PEX19 peptic peptides plotted over time. PEX19 monomer is colored in blue (♦), PEX19:PEX3 complex in red (x). (TIF) [file pone.0103101.s001.tif]

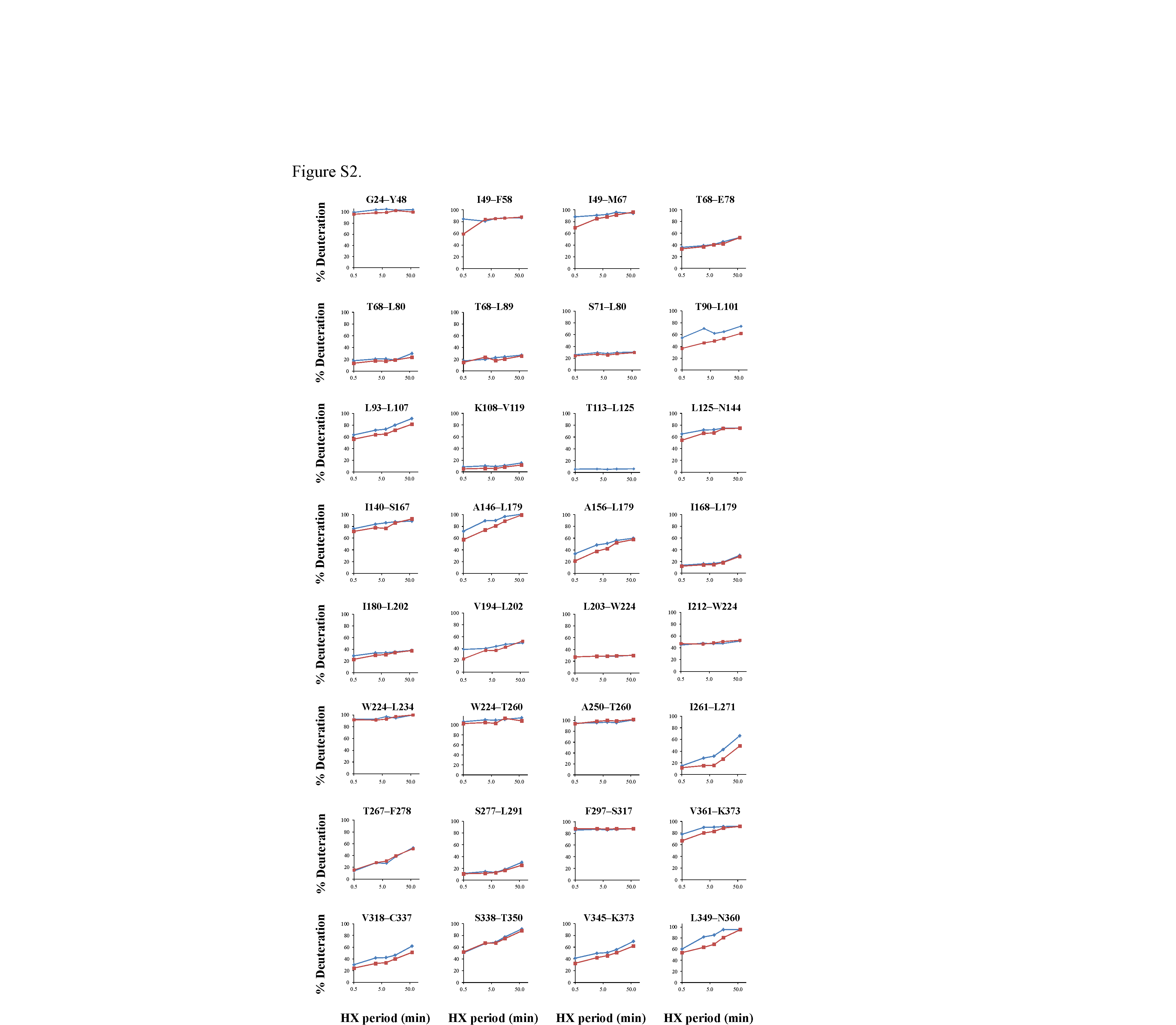

Supplement: Figure S2 — Relative deuterium uptake of PEX3 peptic peptides plotted over time. PEX3 monomer is indicated in blue (♦), PEX3 bound to full-length PEX19 in red (▪). (TIF) [file pone.0103101.s002.tif]
